# Endoscopic closure of a challenging gastrobronchial fistula following esophagectomy: an effective and accessible technique

**DOI:** 10.1055/a-2520-1036

**Published:** 2025-02-11

**Authors:** Xuan Cheng, Huige Wang, Xuanjiang Zhao, Dan Liu

**Affiliations:** 1 191599 Department of Gastroenterology and Hepatology, The First Affiliated Hospital of Zhengzhou University, Zhengzhou, China

A 59-year-old man, who had undergone esophagectomy and gastric conduit reconstruction 5 years previously, underwent endoscopic submucosal dissection (ESD) to remove a submucosal tumor from the gastric conduit. Subsequently, the patient experienced coughing during liquid intake and a mild fever. Thoracic computed tomography (CT) revealed infiltration in the lower lobe of the right lung with a pleural effusion, suggestive of a gastrobronchial fistula on the right side ( Fig. 1 ). An esophagogram confirmed the presence of a leak from the reconstructed gastric conduit ( Fig. 2 ). Upper gastrointestinal endoscopy revealed an unresolved post-ESD ulcer, alongside multiple sinuses, with pus and air bubbles at the base. After repeated irrigation had been performed, the base was exposed, revealing several fistulas, which appeared to communicate with the bronchus ( Fig. 3 **a** ). After informed consent had been obtained, endoscopic closure was selected as the treatment approach ( Video 1 ).

**Fig. 1:**
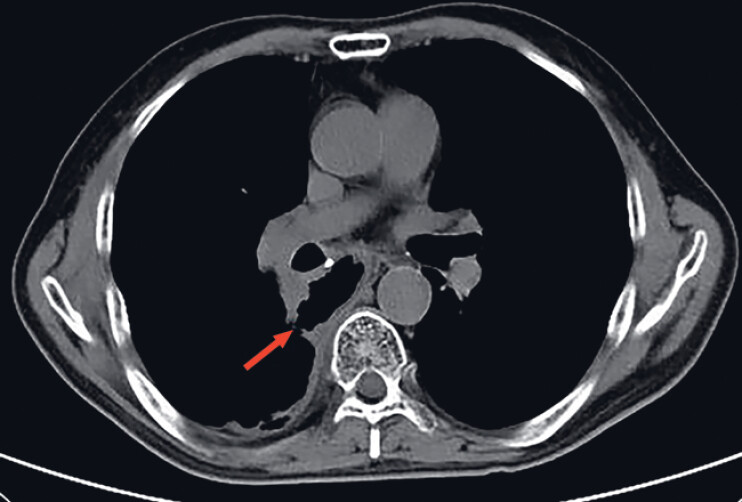
Thoracic computed tomography image showing a right-sided gastrobronchial fistula.

**Fig. 2:**
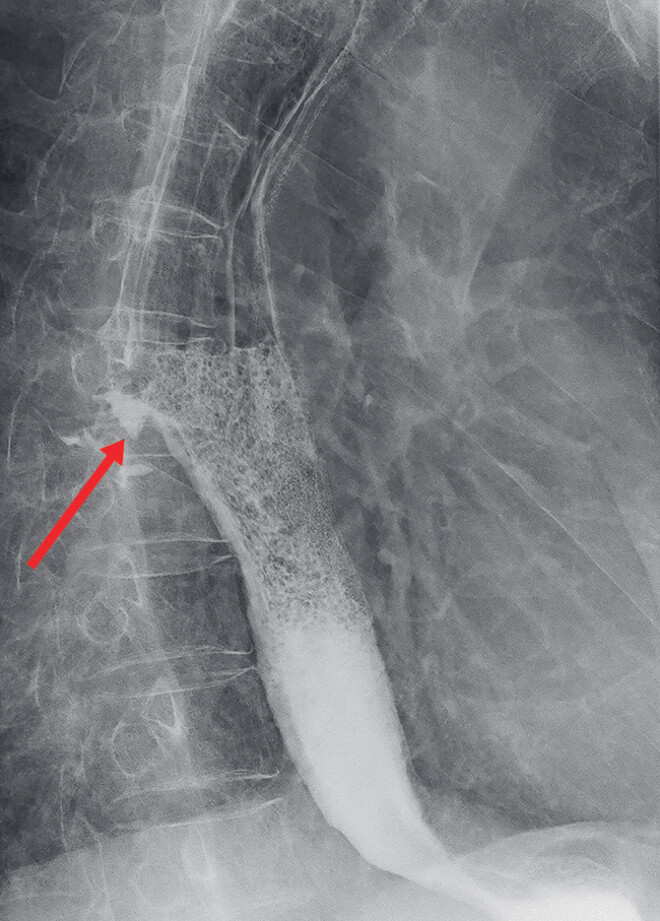
Esophagogram confirming the presence of a leak in the reconstructed gastric conduit.

**Fig. 3:**
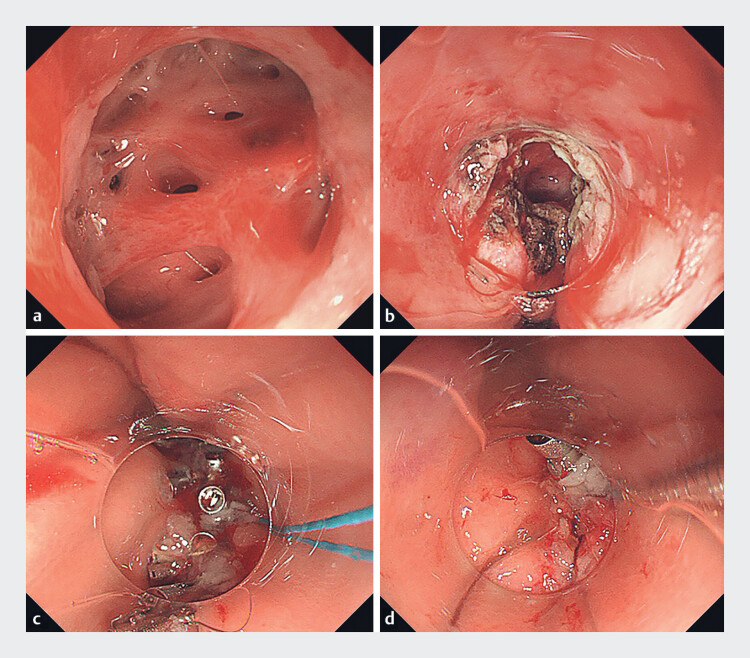
Endoscopic images showing: **a** the appearance of the defect after endoscopic submucosal dissection; **b** electrocoagulation of the defect’s edges; **c** placement of through-the-scope endoclips; **d** release of the nylon cord.

Endoscopic closure of a challenging gastrobronchial fistula following esophagectomy using nylon cord-assisted endoclip closure: an effective and accessible technique.Video 1

Initially, the fistula and surrounding mucosa were treated using electrocoagulation forceps to prevent scarring along the edges, which could have interfered with clipping and wound healing ( Fig. 3 **b** ). Subsequently, a nylon cord was anchored to the opposing margins of the defect using double endoclips and tightened to approximate the edges of the defect. This cord served as traction, facilitating the placement of additional clips, with through-the-scope endoclips then applied sequentially along both sides of the nylon cord, following the longitudinal axis of the stomach ( Fig. 3 **c** ). Finally, the nylon cord was released ( Fig. 3 **d** ). A decompression tube was inserted into the gastric conduit. By postoperative day 3, the esophagogram was showing no extravasation of contrast and the patient resumed oral intake without symptoms. At 3-month follow-up, the patient remained asymptomatic.

Gastrobronchial leakage in the gastric conduit following esophagectomy is a rare and complex complication to manage 1 . The nylon cord-assisted endoclip closure technique has been demonstrated to be a straightforward and effective method for addressing challenging gastrobronchial fistulas without the requirement for additional costly equipment 2 3 .

Endoscopy_UCTN_Code_TTT_1AO_2AI

## Notes

Endoscopy E-Videos https://eref.thieme.de/e-videos
*E-Videos* is an open access online section of the journal *Endoscopy* , reporting on interesting cases and new techniques in gastroenterological endoscopy. All papers include a high-quality video and are published with a Creative Commons CC-BY license. Endoscopy E-Videos qualify for HINARI discounts and waivers and eligibility is automatically checked during the submission process. We grant 100% waivers to articles whose corresponding authors are based in Group A countries and 50% waivers to those who are based in Group B countries as classified by Research4Life (see: https://www.research4life.org/access/eligibility/ ). This section has its own submission website at https://mc.manuscriptcentral.com/e-videos .
